# Different weak inter­actions in the crystals of three isomeric (*E*)-*N*-methyl-*N*′-(nitro­benzyl­idene)-2-(thio­phen-2-yl)acetohydrazides

**DOI:** 10.1107/S2056989016016856

**Published:** 2016-11-01

**Authors:** Laura N. F. Cardoso, Thais C. M. Noguiera, Carlos R. Kaiser, James L. Wardell, Marcus V. N. de Souza, Shaun T. Lancaster, William T. A. Harrison

**Affiliations:** a Fundação Oswaldo Cruz, Instituto de Tecnologia em Fármacos–FarManguinhos, Rua Sizenando Nabuco, 100, Manguinhos, 21041-250 Rio de Janeiro, Brazil; b Instituto de Química, Universidade Federal do Rio de Janeiro, Cidade Universitária, Rio de Janeiro, Brazil; c Department of Chemistry, University of Aberdeen, Meston Walk, Aberdeen AB24 3UE, Scotland

**Keywords:** crystal structure, carbohydrazide, methyl­ation, weak hydrogen bonds

## Abstract

The crystal structures of three isomeric (*E*)-*N*-methyl-*N*′-(nitro­benzyl­idene)-2-(thio­phen-2-yl)acetohydrazides (formula C_14_H_13_N_3_O_3_S) are described, with the nitro group in *ortho*, *meta* and *para* positions in the benzene ring. In each crystal structure, mol­ecules are linked by various weak inter­actions (C—H⋯O and C—H⋯π bonds, and π–π stacking), leading to three-dimensional networks in each case, but with little similarity between them.

## Abstract 2

Three isomeric methyl­ated 2-(thio­phen-2-yl)acetohydrazides show little consistency in the pattern of weak (C—H⋯O, C—H⋯π and π–π) inter­actions in their crystal structures.

## Chemical context   

Our ongoing inter­est in the biological activities and structural chemistry of heterocyclic compounds have led us to investigate compounds containing a thio­phene ring system. We have reported the syntheses and anti-TB activities of acetamido derivatives, 2-(*R*,*R*′NCOCH_2_)-thio­phene (de Souza *et al.*, 2008 ▸) and more recently thienyl acetohydrazide derivatives, 2-(ArCH=N—NHCOCH_2_)-thio­phene (Cardoso *et al.*, 2014 ▸). We have followed up this study with work on (*E*)-*N*-methyl-*N*′-aryl­idene-2-(thio­phen-2-yl)acetohydrazides. The anti-TB activities of these compounds will be reported elsewhere: here, we present the crystal structures of three isomeric derivatives in this family bearing a nitro group on the aromatic ring, *viz.* (*E*)-*N*-methyl-*N*′-(2-nitro­nitro­benzyl­idene)-2-(thio­phen-2-yl)acetohydrazide, (I), (*E*)-*N*-methyl-*N*′-(3-nitro­nitro­benz­yl­idene)-2-(thio­phen-2-yl)acetohydrazide, (II), and (*E*)-*N*-methyl-*N*′-(4-nitro­nitro­benzyl­idene)-2-(thio­phen-2-yl)acetohydrazide, (III).
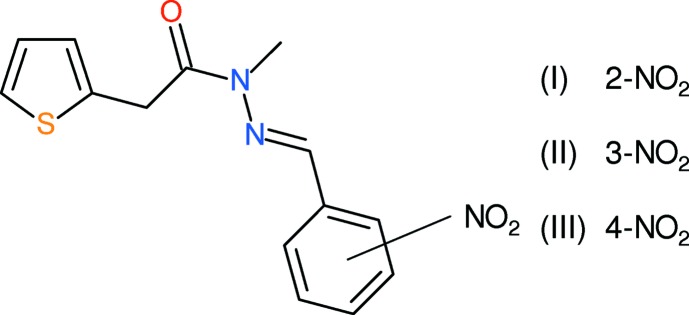


## Structural commentary   

**Figure 1:**
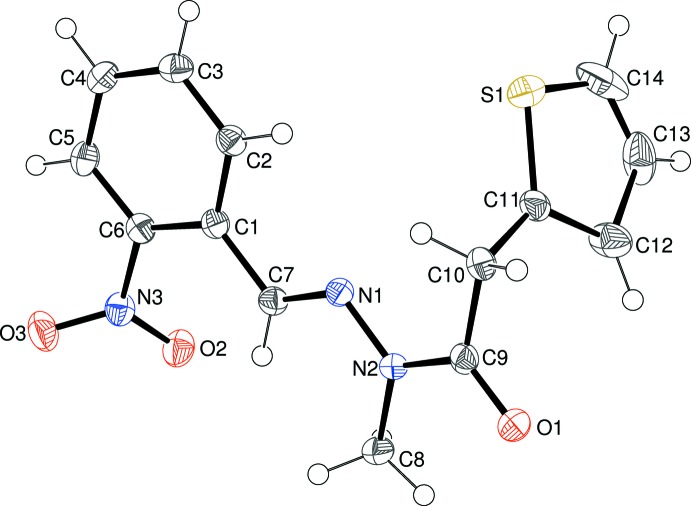
The mol­ecular structure of (I), showing 50% displacement ellipsoids. Only the major orientation of the thio­phene ring is shown.

The mol­ecular structure of (I) is shown in Fig. 1 ▸, which confirms that methyl­ation has occurred at N2. The thio­phene ring (S1/C11–C14) shows ‘flip’ disorder (compare, for example, Sonar *et al.*, 2005 ▸; Wagner *et al.*, 2006 ▸) over two conformations rotated by ∼180° about the C10—C11 bond in a 0.671 (2):0.329 (2) ratio. The dihedral angle between the thio­phene ring and the C1–C6 benzene ring is 77.22 (6)°. The *ortho*-N3/O1/O2 nitro group deviates from the mean plane of its attached benzene ring by 43.61 (5)°: this substantial twist can in part be ascribed to steric reasons. The central CH=N—N(CH_3_)—C(=O)—CH_2_ fragment in (I) is approximately planar (r.m.s. deviation = 0.032 Å) and subtends dihedral angles of 6.39 (5) and 83.61 (6)° with the benzene and thio­phene rings, respectively. Thus, the major twist in the molecule occurs about the C9—C10 bond [N2—C9—C10—C11 = −81.73 (18)°], giving the mol­ecule an approximate overall L-shape. The N1—N2 bond length of 1.3725 (18)° is shorter than the reference value of ∼1.41 Å for an N—N single bond and the C9—N2 amide bond of 1.377 (2) Å is somewhat lengthened: these distance data suggest significant delocalization of electrons over the methyl­idene—acetohydrazide grouping.

**Figure 2:**
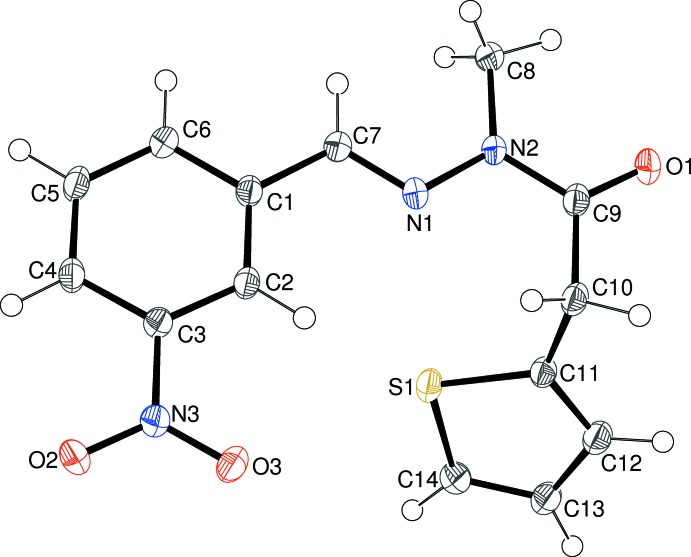
The mol­ecular structure of (II), showing 50% displacement ellipsoids.

The mol­ecular structure of (II) can be seen in Fig. 2 ▸; again the methyl­ation of N2 has occurred as expected but this time the S1/C11–C14 thio­phene ring shows no detectable sign of disorder [C11—S1—C14 = 92.35 (6)°]. The dihedral angle between the thio­phene ring and the C1–C6 benzene ring is 60.17 (4)°. The *meta*-N3/O1/O2 nitro group is almost coplanar with its attached benzene ring [dihedral angle = 1.96 (2)°]. The almost planar central methyl­idene–acetohydrazide grouping in (II) (r.m.s. deviation = 0.006 Å) subtends dihedral angles of 7.27 (7)° with the benzene ring and 61.67 (4)° with the thio­phene ring. As in (I), the major twist occurs about C9—C10 [N2—C9—C10—C11 = 85.18 (14)°], again giving the mol­ecule an approximate overall L-shape. The N1—N2 and C9—N2 bond lengths in (II) are 1.3747 (14) and 1.3776 (15) Å, respectively, which again can be ascribed to delocalization.

**Figure 3:**
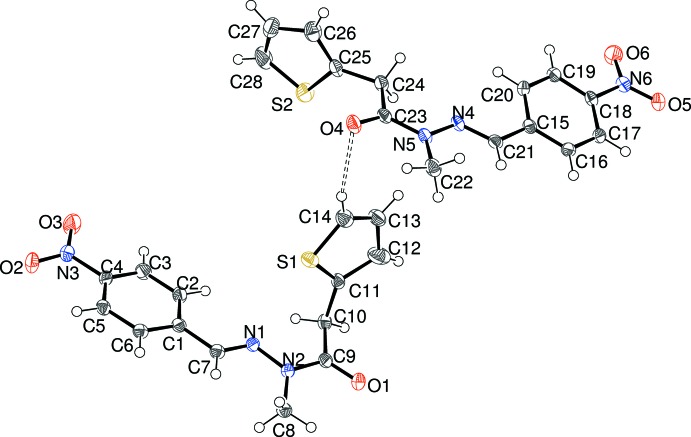
The mol­ecular structure of (III), showing 50% displacement ellipsoids. Only the major orientation of the thio­phene ring is shown.

**Figure 4:**
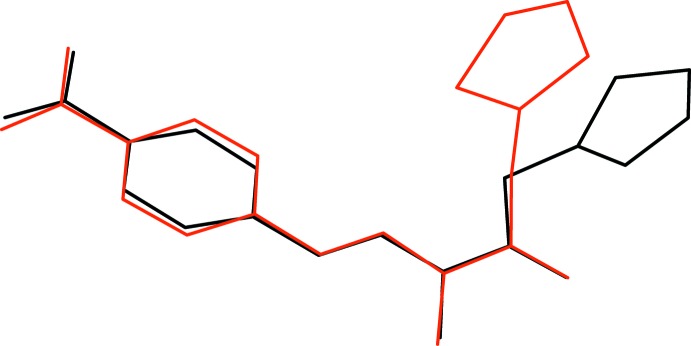
Overlay plot of the N1 (red) and N4 (black) mol­ecules in (III).

Compound (III) crystallizes with two mol­ecules (methyl­ated at N2 and N5) in the asymmetric unit with different conformations (Fig. 3 ▸); in both mol­ecules the thio­phene ring is rotationally disordered [major/minor disorder components = 0.673 (3):0.327 (3) for the S1 ring and 0.832 (3):0.168 (3) for the S2 ring. In the S1 mol­ecule, the dihedral angles between the benzene ring ‘*A*’, thio­phene ring ‘*B*’ and CH=N—N(CH_3_)—C(=O)—CH_2_ fragment ‘*C*’ (r.m.s. deviation = 0.034 Å), are *A*/*B* = 79.36 (6), *A*/*C* = 12.75 (12) and *B*/*C* = 69.60 (6)°. Equivalent dihedral-angle data for the S2 mol­ecule are 88.23 (6), 15.51 (13) and 82.51 (6)°, respectively. The *para*-nitro group is twisted from its attached ring by 9.2 (3) (S1 mol­ecule) and 8.8 (3)° (S2 molecule). The dihedral angles are broadly similar but even so, the two mol­ecules have different conformations (Fig. 4 ▸) as indicated by the N2—C9—C10—C11 and N5—C23—C24—C25 torsion angles of 91.7 (2) and 171.09 (17), respectively. Bond-length data [N1—N2 = 1.373 (2), C9—N2 = 1.380 (3), N4—N5 = 1.368 (2) and C23—N5 = 1.384 (2) Å] are consistent between the mol­ecules and with the equivalent data for (I) and (II).

## Supra­molecular features   

**Figure 5:**
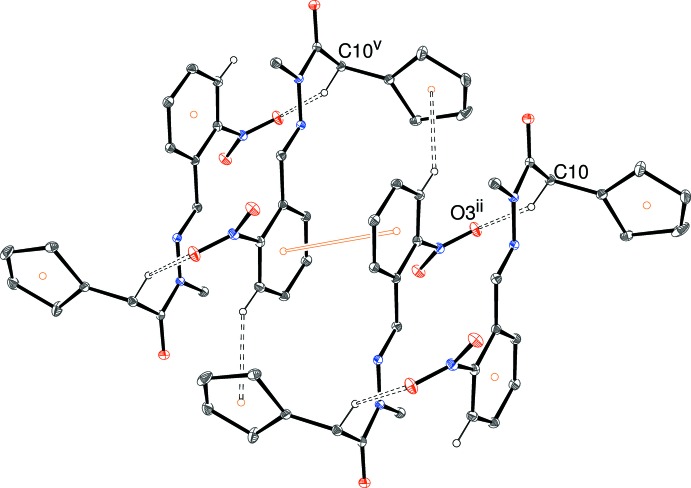
Fragment of a [100] hydrogen-bonded chain in the crystal of (I). [Symmetry codes: (ii) −*x*, 1 − *y*, 1 − *z*; (v) 1 + *x*, *y*, *z*.] All H atoms not involved in hydrogen bonds have been omitted for clarity.

**Figure 6:**
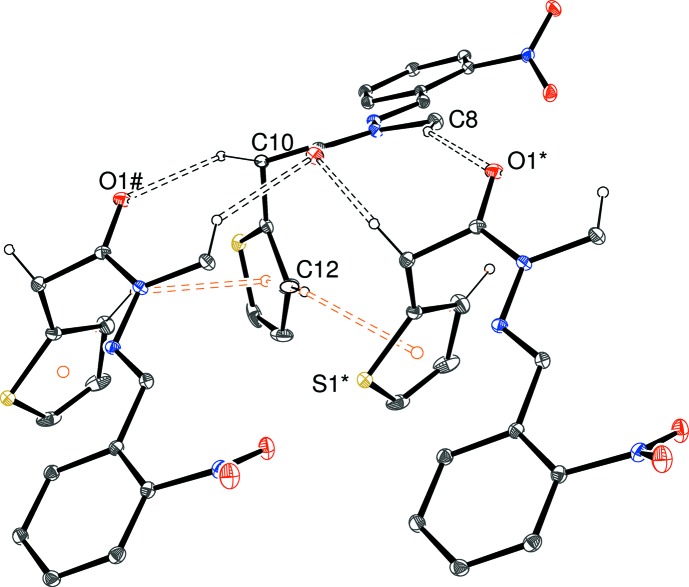
Fragment of an [001] hydrogen-bonded chain in the crystal of (I). [Symmetry codes: (*) *x*, 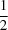 − *y*, *z* − 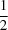; (#) *x*, 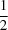 − *y*, 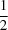 + *z*.] All H atoms not involved in hydrogen bonds have been omitted for clarity.

The packing in (I) can be decomposed into two different chains: in the first of these (Fig. 5 ▸), inversion dimers (about the point 0, 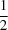, 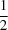 for the asymmetric mol­ecule) linked by pairs of C10—H10a⋯O3 hydrogen bonds (Table 1 ▸) generate 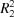
(20) loops. These dimers are complemented by inversion-related pairs of C5—H5⋯*Cg*1 (where *Cg*1 is the centroid of the thio­phene ring) bonds; this second inversion dimer (about 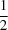, 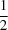, 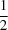) is reinforced by an aromatic π–π stacking inter­action involving the C1–C6 benzene rings [centroid separation = 3.7118 (9) Å; slippage = 1.27 Å]. Together, the C—H⋯O dimers and the C—H⋯π + π–π dimers alternate in [100] chains. In the second one-dimensional motif, the C8, C10—H10b and C12 bonds combine together to generate [001] chains (Fig. 6 ▸) in which the carbonyl O1 atom accepts hydrogen bonds from two adjacent mol­ecules to generate 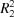
(9) loops. The cohesion of the chain is reinforced by a C—H⋯π inter­action from one thio­phine ring to the next: the dihedral angle between two adjacent rings in the chain is 73.32 (4)°. Taken together, the [100] and [001] chains combine together to generate a three-dimensional network.

**Figure 7:**
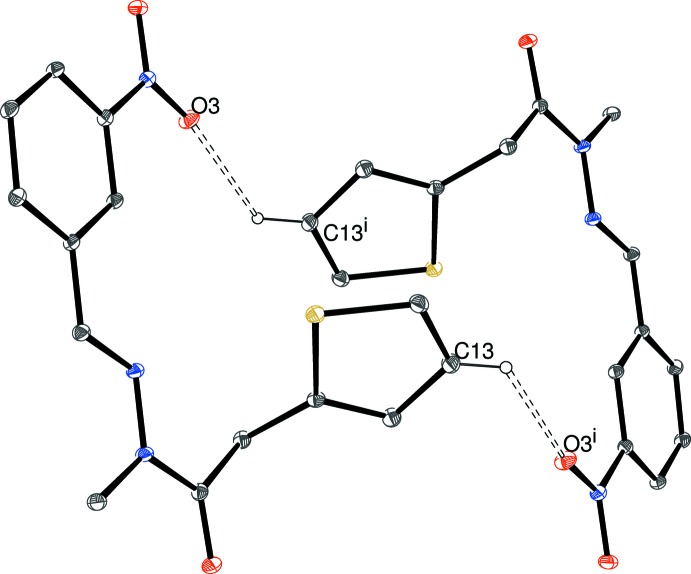
Inversion dimer in the crystal of (II) linked by a pair of C—H⋯O hydrogen bonds. [Symmetry code: (i) 2 − *x*, 1 − *y*, 1 − *z*.] All H atoms not involved in hydrogen bonds have been omitted for clarity.

The packing in (II) features four C—H⋯O inter­actions (Fig. 7 ▸, Table 2 ▸); the C13 bond (Fig. 2 ▸) generates 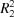
(28) loops and the C7 bond leads to *C*(7) chains propagating in [010]. The two C8 (methyl-group) bonds lead to (101) sheets. Taken together, these inter­actions lead to a three-dimensional network of mol­ecules in the crystal. There are no C—H⋯π or π–π stacking inter­actions in (II).

**Figure 8:**
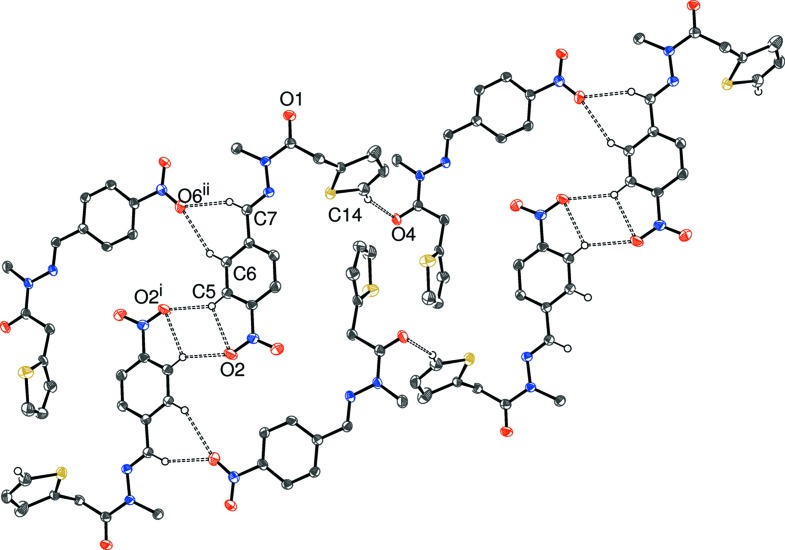
Fragment of a [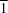
01] hydrogen-bonded chain in the crystal of (III). [Symmetry codes: (i) 2 − *x*, −*y*, −*z*; (ii) *x* + 1, *y*, *z* − 1.] All H atoms not involved in hydrogen bonds have been omitted for clarity.

The packing for (III) can be visualized in terms of two different chains. The first of these (Table 3 ▸, Figs. 8 ▸ and 9 ▸), which involves the four C—H donor groups of the C1-mol­ecule, is built up from inversion dimers (about the point 1,0,0 for the asymmetric mol­ecule) of C1-mol­ecules linked by pairs of C5—H5⋯O2 hydrogen bonds, which generate 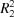
(8) loops. The C6—H6 and C7—H7 groups link to the same acceptor atom (O6; part of the C15 mol­ecule), to generate an 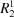
(6) loop. Finally, C14—H14 (part of the thio­phene ring) forms a bond to O4 in another nearby C15-mol­ecule. The C15 mol­ecules in turn link to further pairs of C1-mol­ecules and hence form [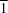
01] chains. The second chain in (III) (Fig. 10) features the donor groups of the C15-mol­ecule; the C17—H17 (to O1) and C20—H20 (to O3) bonds arise from different sides of the benzene ring and both the acceptor atoms are parts of C1-mol­ecules: the end result is a [2
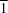
0] chain of alternating C1- and C15-mol­ecules. Taken together, a complex three-dimensional network arises, which may be consolidated by a pair of weak C—H⋯π inter­actions arising from methyl groups, assuming that the H atoms in question have been reliably located.

## Database survey   

A survey of the Cambridge Structural Database (V5.37, last update May 2016; Groom *et al.*, 2016 ▸) for the common central –CH=N—N(CH_3_)—C(=O)—CH_2_– fragment of the title compounds revealed just three matches, *viz.* FOTMUX (Ramirez *et al.*, 2009*a* ▸), KULREP (Ramirez *et al.*, 2009*b* ▸) and OFEBIL (Cao *et al.*, 2007 ▸). FOTMUX is an inter­esting binuclear copper complex but none of these materials have a close relationship to the isomeric compounds reported here.

## Synthesis and crystallization   

The appropriate derivative (Cardoso *et al.*, 2014 ▸) of (1) (0.2 g, 1.0 equivalent) was suspended in acetone (5.0 ml) and potassium carbonate (4.0 equivalents) was added. The reaction mixture was stirred at room temperature for 30 min and methyl iodide (4.0 equivalents) was added. The reaction mixture was maintained at 313 K, until thin-layer chromatography indicated that the reaction was complete. The reaction mixture was rotary evaporated to leave a residue, which was dissolved in water (20.0 ml) and extracted with ethyl acetate (3 × 10.0 ml). The organic phases were combined, dried with anhydrous MgSO_4_, filtered and then evaporated at reduced pressure. The crystals used for intensity data collection were recrystallized from ethanol solution.

(*E*)-*N*-Methyl-*N*′-(2-nitro­phenyl­methyl­idene)-2-(thio­phen-2-­yl)acetohydrazide, (I); yield: 57%; yellow solid; m.p. 366–367 K. ^1^H NMR (400 MHz, DMSO): δ 8.21 (1H; *s*; N=CH), 8.12 (1H; *dd*; *J*_HH_ = 8.0 and 1.2 Hz; H-11′), 8.04 (1H; *dd*; *J*_HH_ = 8.4 and 0.8 Hz; H-8′), 7.83–7.80 (1H; *m*; H-10′), 7.69–7.67 (1H; *m*; H-9′), 7.37 (1H; *dd*; *J*_HH_ = 4.8 and 1.6 Hz; H-5) 6.96–6.94 (2H; *m*; H-3 and H-4), 4.34 (2H; *s*; CH_2_), 3.32 (3H; *s*; N—CH_3_). ^13^C NMR (125 MHz; DMSO): δ 171.0 (C=O), 148.3 (C-7′), 136.8 (N=CH), 136.1 (C-2), 133.4 (C-10′), 130.4 (C-9′), 128.8 (C-11′), 128.3 (C-6′), 126.8 (C-3), 126.5 (C-4), 125.2 (C-5), 124.5 (C-8′), 33.9 (N-CH_3_), 28.1 (CH_2_). MS/ESI: [*M* + Na]: 326. IR ν_max_ (cm^−1^; KBr pellet): 1681 (C=O); 3088 (N-CH_3_).

(*E*)-*N*-Methyl-*N*′-(3-nitro­phenyl­methyl­idene)-2-(thio­phen-2-­yl)acetohydrazide, (II); yield: 73%; yellow solid; m.p. 378–383 K. 1H NMR (400 MHz, DMSO): δ 8.61 (1H; *s*; N=CH), 8.29–8.25 (2H; *m*; H-11′ and H-9′), 8.17 (1H; *s*; H-7′), 7.79–7.75 (1H; *m*; H-10′), 7.37–7.35 (1H; *m*; H-5), 7.00–6.99 (1H; *m*; H-4) 6.96–6.94 (1H; *m*; H-3), 4.40 (2H; *s*; CH_2_), 3.35 (3H; *s*; N-CH_3_). ^13^C NMR (125 MHz; DMSO) δ: 170.9 (C=O), 148.2 (C-8′), 138.6 (N=CH), 136.9 (C-2), 136.5 (C-6′), 132.8 (C-11′), 130.4 (C-10′), 126.7 (C-9′), 126.6 (C-3), 125.2 (C-4), 123.9 (C-5), 121.6 (C-7′), 34.3 (N-CH_3_), 28.2 (CH_2_). MS/ESI: [*M* + Na]: 326. IR ν_max_ (cm^−1^; KBr pellet): 1668 (C=O); 2962 (N—CH_3_).

(*E*)-*N*-Methyl-*N*′-(4-nitro­phenyl­methyl­idene)-2-(thio­phen-2-­yl)acetohydrazide, (III); yield: 55%; yellow solid; m.p. 428–433 K. ^1^H NMR (400 MHz; DMSO) δ: 8.32 (2H; d; *J*_HH_ = 8.8 Hz; H-8′ and H-10′), 8.13 (1H; *s*; N=CH), 8.07 (2H; d; *J*_HH_ = 8.8 Hz; H-7′ and H-11′), 7.36 (1H; *dd*; *J*_HH_ = 4.8 and 1.2 Hz H-5), 7.00-6.99 (1H; *m*; H-3), 6.96-6.94 (1H; *m*; H-4), 4.41 (2H; *s*; CH_2_), 3.36 (3H; *s*; N-CH_3_). ^13^C NMR (125 MHz; DMSO) δ: 171.0 (C=O), 147.6 (C-9′), 140.9 (N=CH), 138.4 (C-6′), 136.8 (C-2), 128.0 (C-3), 126.8 (C-4), 126.5 (C-5), 125.2 (C-7′ and C-11′), 124.0 (C-C-8′ and C-10′), 34.2 (N-CH_3_), 28.3 (CH_2_). MS/ESI: [*M* + Na]: 326. IR ν_max_ (cm^−1^; KBr pellet): 1678 (C=O); 3101 (N-CH_3_).

## Refinement   

Crystal data, data collection and structure refinement details are summarized in Table 4 ▸. The H atoms were placed geometrically (C—H = 0.95–1.00 Å) and refined as riding atoms. The constraint *U*_iso_(H) = 1.2*U*_eq_(carrier) or 1.5*U*_eq_(meth­yl) was applied in all cases. The methyl group was allowed to rotate, but not to tip, to best fit the electron density (AFIX 137 instruction). In each case, this group rotated from its initial orientation to minimize steric inter­action with atom H7; the final orientation leads to a short C8—H⋯O1 intra­molecular contact but we do not regard this as a bond. The thio­phene rings in (I) and (III) show ‘flip’ rotational disorder.

## Supplementary Material

Crystal structure: contains datablock(s) I, II, III, global. DOI: 10.1107/S2056989016016856/hg5478sup1.cif

Structure factors: contains datablock(s) I. DOI: 10.1107/S2056989016016856/hg5478Isup2.hkl

Structure factors: contains datablock(s) II. DOI: 10.1107/S2056989016016856/hg5478IIsup3.hkl

Structure factors: contains datablock(s) III. DOI: 10.1107/S2056989016016856/hg5478IIIsup4.hkl

Supporting information file. DOI: 10.1107/S2056989016016856/hg5478Isup5.cml

Supporting information file. DOI: 10.1107/S2056989016016856/hg5478IIsup6.cml

Supporting information file. DOI: 10.1107/S2056989016016856/hg5478IIIsup7.cml

CCDC references: 1510866, 1510865, 1510864

Additional supporting information:  crystallographic information; 3D view; checkCIF report

## Figures and Tables

**Figure 9:**
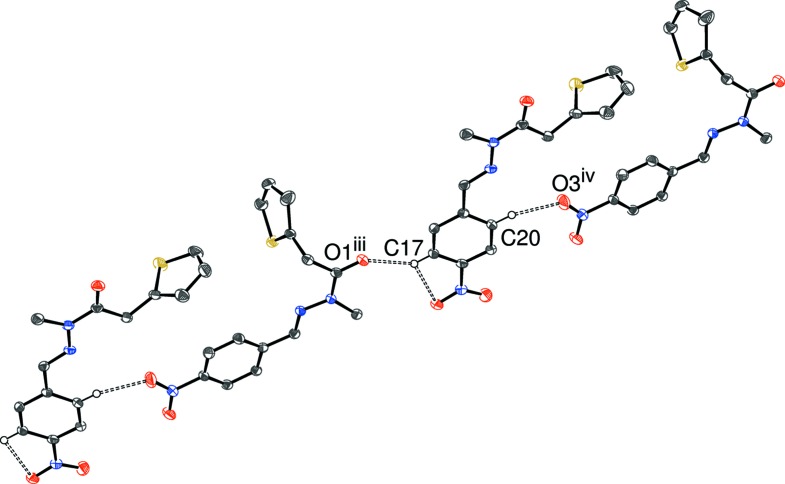
Fragment of a [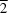
10] hydrogen-bonded chain in the crystal of (III). [Symmetry codes: (iii) 1 − *x*, 1 − *y*, 1 − *z*; (iv) 1 − *x*, −*y*, 1 − *z*.] All H atoms not involved in hydrogen bonds have been omitted for clarity.

**Table 1:** Hydrogen-bond geometry (Å, °) for (I) *Cg*1 is the centroid of the thiophene ring.

*D*—H⋯*A*	*D*—H	H⋯*A*	*D*⋯*A*	*D*—H⋯*A*
C8—H8*C*⋯O1^i^	0.98	2.49	3.293 (2)	139
C10—H10*A*⋯O3^ii^	0.99	2.55	3.386 (2)	142
C10—H10*B*⋯O1^iii^	0.99	2.52	3.439 (2)	154
C5—H5⋯*Cg*1^iv^	0.95	2.86	3.7212 (18)	151
C12—H12⋯*Cg*1^i^	0.95	2.85	3.5930 (13)	136

Symmetry codes: (i) 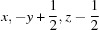; (ii) 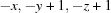; (iii) 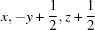; (iv) 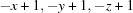.

**Table 2:** Hydrogen-bond geometry (Å, °) for (II)

*D*—H⋯*A*	*D*—H	H⋯*A*	*D*⋯*A*	*D*—H⋯*A*
C7—H7⋯O2^i^	0.95	2.39	3.2879 (16)	157
C8—H8*B*⋯O2^ii^	0.98	2.50	3.3468 (16)	144
C8—H8*C*⋯O3^iii^	0.98	2.52	3.4356 (17)	156
C13—H13⋯O3^iv^	0.95	2.52	3.1874 (16)	127

Symmetry codes: (i) 
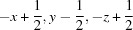
; (ii) 
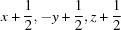
; (iii) 
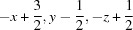
; (iv) 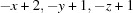.

**Table 3:** Hydrogen-bond geometry (Å, °) for (III) *Cg*6 is the centroid of the C15–C20 ring.

*D*—H⋯*A*	*D*—H	H⋯*A*	*D*⋯*A*	*D*—H⋯*A*
C5—H5⋯O2^i^	0.95	2.48	3.312 (3)	147
C6—H6⋯O6^ii^	0.95	2.56	3.412 (2)	149
C7—H7⋯O6^ii^	0.95	2.41	3.281 (3)	153
C14—H14⋯O4	0.95	2.55	3.464 (3)	160
C17—H17⋯O1^iii^	0.95	2.43	3.104 (2)	128
C20—H20⋯O3^iv^	0.95	2.33	3.176 (2)	147
C8—H8*B*⋯*Cg*6^v^	0.98	2.77	3.634 (2)	147
C24—H24*A*⋯*Cg*6^vi^	0.98	2.77	3.628 (2)	145

Symmetry codes: (i) 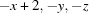; (ii) 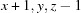; (iii) 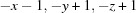; (iv) 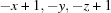; (v) 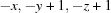; (vi) 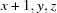.

**Table 4:** Experimental details

	(I)	(II)	(III)
Crystal data			
Chemical formula	C_14_H_13_N_3_O_3_S	C_14_H_13_N_3_O_3_S	C_14_H_13_N_3_O_3_S
*M*_r_	303.33	303.33	303.33
Crystal system, space group	Monoclinic, *P*2_1_/*c*	Monoclinic, *P*2_1_/*n*	Triclinic, *P* 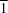
Temperature (K)	100	100	100
*a*, *b*, *c* (Å)	7.3989 (5), 24.4910 (17), 7.7126 (5)	5.6629 (4), 15.6864 (11), 15.2842 (11)	6.1893 (4), 12.9177 (9), 17.3828 (12)
α, β, γ (°)	90, 96.022 (2), 90	90, 93.3800 (18), 90	93.995 (7), 90.386 (6), 95.963 (7)
*V* (Å^3^)	1389.86 (16)	1355.34 (17)	1378.77 (16)
*Z*	4	4	4
Radiation type	Mo *K*α	Mo *K*α	Mo *K*α
μ (mm^−1^)	0.25	0.25	0.25
Crystal size (mm)	0.08 × 0.07 × 0.03	0.22 × 0.17 × 0.12	0.20 × 0.18 × 0.16

Data collection			
Diffractometer	Rigaku Mercury CCD	Rigaku Mercury CCD	Rigaku Mercury CCD
No. of measured, independent and observed [*I* > 2σ(*I*)] reflections	9379, 3157, 2439	9365, 3110, 2757	18534, 6279, 4868
*R*_int_	0.040	0.031	0.078
(sin θ/λ)_max_ (Å^−1^)	0.648	0.649	0.649

Refinement			
*R*[*F* ^2^ > 2σ(*F* ^2^)], *wR*(*F* ^2^), *S*	0.041, 0.106, 1.05	0.034, 0.096, 1.08	0.058, 0.166, 1.10
No. of reflections	3157	3110	6279
No. of parameters	192	191	383
H-atom treatment	H-atom parameters constrained	H-atom parameters constrained	H-atom parameters constrained
Δρ_max_, Δρ_min_ (e Å^−3^)	0.45, −0.35	0.30, −0.28	0.67, −0.61

Computer programs: *CrystalClear* (Rigaku, 2012 ▸), *SHELXS97* (Sheldrick, 2008 ▸), *SHELXL2014* (Sheldrick, 2015 ▸), *ORTEP-3 for Windows* (Farrugia, 2012 ▸) and *publCIF* (Westrip, 2010 ▸).

## supplementary crystallographic information

### (I) (E)-N-Methyl-N'-(2-nitrobenzylidene)-2-(thiophen-2-yl)acetohydrazide Crystal data

**Table:**

C_14_H_13_N_3_O_3_S	*F*(000) = 632
*M**_r_* = 303.33	*D*_x_ = 1.450 Mg m^−^^3^
Monoclinic, *P*2_1_/*c*	Mo *K*α radiation, λ = 0.71073 Å
*a* = 7.3989 (5) Å	Cell parameters from 9051 reflections
*b* = 24.4910 (17) Å	θ = 2.5–27.5°
*c* = 7.7126 (5) Å	µ = 0.25 mm^−^^1^
β = 96.022 (2)°	*T* = 100 K
*V* = 1389.86 (16) Å^3^	Block, pale yellow
*Z* = 4	0.08 × 0.07 × 0.03 mm

### (I) (E)-N-Methyl-N'-(2-nitrobenzylidene)-2-(thiophen-2-yl)acetohydrazide Data collection

**Table:**

Rigaku Mercury CCD diffractometer	*R*_int_ = 0.040
ω scans	θ_max_ = 27.4°, θ_min_ = 2.8°
9379 measured reflections	*h* = −9→9
3157 independent reflections	*k* = −31→28
2439 reflections with *I* > 2σ(*I*)	*l* = −9→9

### (I) (E)-N-Methyl-N'-(2-nitrobenzylidene)-2-(thiophen-2-yl)acetohydrazide Refinement

**Table:**

Refinement on *F*^2^	Primary atom site location: structure-invariant direct methods
Least-squares matrix: full	Hydrogen site location: inferred from neighbouring sites
*R*[*F*^2^ > 2σ(*F*^2^)] = 0.041	H-atom parameters constrained
*wR*(*F*^2^) = 0.106	*w* = 1/[σ^2^(*F*_o_^2^) + (0.0494*P*)^2^ + 0.321*P*] where *P* = (*F*_o_^2^ + 2*F*_c_^2^)/3
*S* = 1.05	(Δ/σ)_max_ = 0.001
3157 reflections	Δρ_max_ = 0.45 e Å^−^^3^
192 parameters	Δρ_min_ = −0.35 e Å^−^^3^
0 restraints	

### (I) (E)-N-Methyl-N'-(2-nitrobenzylidene)-2-(thiophen-2-yl)acetohydrazide Special details

**Table:**

Geometry. All esds (except the esd in the dihedral angle between two l.s. planes) are estimated using the full covariance matrix. The cell esds are taken into account individually in the estimation of esds in distances, angles and torsion angles; correlations between esds in cell parameters are only used when they are defined by crystal symmetry. An approximate (isotropic) treatment of cell esds is used for estimating esds involving l.s. planes.

### (I) (E)-N-Methyl-N'-(2-nitrobenzylidene)-2-(thiophen-2-yl)acetohydrazide Fractional atomic coordinates and isotropic or equivalent isotropic displacement parameters (Å^2^)

**Table:**

	*x*	*y*	*z*	*U*_iso_*/*U*_eq_	Occ. (<1)
C1	0.2369 (2)	0.49283 (6)	0.4509 (2)	0.0194 (3)	
C2	0.2812 (2)	0.50348 (7)	0.6291 (2)	0.0217 (3)	
H2	0.2668	0.4754	0.7116	0.026*	
C3	0.3455 (2)	0.55416 (7)	0.6873 (2)	0.0242 (4)	
H3	0.3774	0.5601	0.8084	0.029*	
C4	0.3637 (2)	0.59635 (7)	0.5702 (2)	0.0244 (4)	
H4	0.4078	0.6310	0.6111	0.029*	
C5	0.3173 (2)	0.58784 (7)	0.3934 (2)	0.0238 (4)	
H5	0.3267	0.6166	0.3122	0.029*	
C6	0.2570 (2)	0.53656 (7)	0.3373 (2)	0.0208 (3)	
C7	0.1640 (2)	0.43927 (6)	0.3917 (2)	0.0203 (3)	
H7	0.1346	0.4320	0.2710	0.024*	
C8	0.0311 (2)	0.34081 (7)	0.2640 (2)	0.0244 (4)	
H8A	0.1427	0.3440	0.2066	0.037*	
H8B	−0.0598	0.3665	0.2108	0.037*	
H8C	−0.0161	0.3035	0.2504	0.037*	
C9	0.0357 (2)	0.31573 (7)	0.5737 (2)	0.0205 (3)	
C10	0.0923 (2)	0.33017 (7)	0.7629 (2)	0.0218 (3)	
H10A	0.0681	0.3693	0.7822	0.026*	
H10B	0.0197	0.3086	0.8391	0.026*	
C11	0.2913 (2)	0.31855 (7)	0.8097 (2)	0.0235 (4)	
C12	0.39380 (13)	0.27005 (4)	0.74418 (13)	0.0357 (3)	0.671 (2)
H12	0.3487	0.2421	0.6658	0.043*	0.671 (2)
S1A	0.39380 (13)	0.27005 (4)	0.74418 (13)	0.0357 (3)	0.329 (2)
C13	0.5816 (3)	0.27681 (9)	0.8322 (3)	0.0432 (5)	
H13	0.6795	0.2541	0.8069	0.052*	
C14	0.6023 (3)	0.31711 (10)	0.9492 (3)	0.0499 (7)	
H14	0.7135	0.3238	1.0191	0.060*	
N1	0.14114 (17)	0.40276 (5)	0.50578 (17)	0.0191 (3)	
N2	0.07069 (18)	0.35331 (5)	0.44877 (16)	0.0194 (3)	
N3	0.21802 (19)	0.52965 (6)	0.14698 (17)	0.0239 (3)	
O1	−0.03469 (16)	0.27167 (5)	0.53215 (15)	0.0262 (3)	
O2	0.27226 (18)	0.48824 (5)	0.07864 (15)	0.0309 (3)	
O3	0.13598 (19)	0.56675 (5)	0.06483 (16)	0.0341 (3)	
S1	0.42291 (8)	0.35327 (2)	0.96161 (7)	0.0275 (2)	0.671 (2)
C12A	0.42291 (8)	0.35327 (2)	0.96161 (7)	0.0275 (2)	0.329 (2)
H12A	0.3995	0.3839	1.0314	0.033*	0.329 (2)

### (I) (E)-N-Methyl-N'-(2-nitrobenzylidene)-2-(thiophen-2-yl)acetohydrazide Atomic displacement parameters (Å^2^)

**Table:**

	*U*^11^	*U*^22^	*U*^33^	*U*^12^	*U*^13^	*U*^23^
C1	0.0174 (7)	0.0205 (8)	0.0205 (7)	0.0011 (6)	0.0027 (6)	0.0006 (6)
C2	0.0215 (8)	0.0229 (8)	0.0207 (7)	−0.0004 (6)	0.0023 (6)	0.0018 (6)
C3	0.0229 (8)	0.0279 (9)	0.0217 (8)	−0.0008 (7)	0.0021 (6)	−0.0041 (7)
C4	0.0238 (8)	0.0198 (8)	0.0301 (9)	−0.0013 (7)	0.0047 (7)	−0.0038 (7)
C5	0.0243 (8)	0.0202 (8)	0.0276 (8)	0.0011 (7)	0.0070 (7)	0.0026 (7)
C6	0.0203 (8)	0.0228 (8)	0.0197 (7)	0.0021 (6)	0.0034 (6)	0.0004 (6)
C7	0.0218 (8)	0.0208 (8)	0.0182 (7)	0.0009 (6)	0.0014 (6)	−0.0006 (6)
C8	0.0296 (9)	0.0220 (8)	0.0207 (8)	−0.0020 (7)	−0.0018 (7)	−0.0023 (6)
C9	0.0162 (7)	0.0198 (8)	0.0251 (8)	0.0014 (6)	0.0005 (6)	0.0014 (6)
C10	0.0233 (8)	0.0217 (8)	0.0205 (7)	0.0009 (7)	0.0026 (6)	0.0032 (6)
C11	0.0251 (9)	0.0235 (9)	0.0210 (7)	−0.0045 (7)	−0.0016 (6)	0.0060 (6)
C12	0.0237 (5)	0.0446 (6)	0.0375 (6)	0.0074 (4)	−0.0027 (4)	−0.0100 (4)
S1A	0.0237 (5)	0.0446 (6)	0.0375 (6)	0.0074 (4)	−0.0027 (4)	−0.0100 (4)
C13	0.0219 (9)	0.0369 (11)	0.0710 (15)	0.0058 (8)	0.0058 (9)	0.0196 (11)
C14	0.0382 (12)	0.0756 (17)	0.0322 (10)	−0.0314 (11)	−0.0139 (9)	0.0259 (11)
N1	0.0174 (6)	0.0181 (7)	0.0214 (6)	0.0002 (5)	0.0005 (5)	−0.0007 (5)
N2	0.0210 (7)	0.0176 (7)	0.0188 (6)	−0.0009 (5)	−0.0020 (5)	−0.0007 (5)
N3	0.0268 (7)	0.0231 (7)	0.0227 (7)	0.0008 (6)	0.0060 (6)	0.0027 (6)
O1	0.0269 (6)	0.0213 (6)	0.0295 (6)	−0.0040 (5)	−0.0008 (5)	0.0013 (5)
O2	0.0457 (8)	0.0252 (6)	0.0228 (6)	0.0053 (6)	0.0083 (5)	−0.0007 (5)
O3	0.0435 (8)	0.0325 (7)	0.0259 (6)	0.0114 (6)	0.0014 (6)	0.0069 (5)
S1	0.0298 (3)	0.0282 (3)	0.0251 (3)	−0.0060 (2)	0.0056 (2)	−0.0042 (2)
C12A	0.0298 (3)	0.0282 (3)	0.0251 (3)	−0.0060 (2)	0.0056 (2)	−0.0042 (2)

### (I) (E)-N-Methyl-N'-(2-nitrobenzylidene)-2-(thiophen-2-yl)acetohydrazide Geometric parameters (Å, º)

**Table:**

C1—C6	1.402 (2)	C9—C10	1.517 (2)
C1—C2	1.404 (2)	C10—C11	1.506 (2)
C1—C7	1.473 (2)	C10—H10A	0.9900
C2—C3	1.387 (2)	C10—H10B	0.9900
C2—H2	0.9500	C11—S1A	1.524 (2)
C3—C4	1.388 (2)	C11—C12	1.524 (2)
C3—H3	0.9500	C11—C12A	1.6748 (17)
C4—C5	1.386 (2)	C11—S1	1.6748 (17)
C4—H4	0.9500	C12—C13	1.490 (2)
C5—C6	1.387 (2)	C12—H12	0.9500
C5—H5	0.9500	S1A—C13	1.490 (2)
C6—N3	1.476 (2)	C13—C14	1.335 (3)
C7—N1	1.278 (2)	C13—H13	0.9500
C7—H7	0.9500	C14—C12A	1.607 (3)
C8—N2	1.4568 (19)	C14—S1	1.607 (3)
C8—H8A	0.9800	C14—H14	0.9500
C8—H8B	0.9800	N1—N2	1.3725 (18)
C8—H8C	0.9800	N3—O2	1.2294 (18)
C9—O1	1.2262 (19)	N3—O3	1.2308 (18)
C9—N2	1.377 (2)	C12A—H12A	0.9500
			
C6—C1—C2	116.18 (15)	C11—C10—H10B	109.5
C6—C1—C7	123.10 (14)	C9—C10—H10B	109.5
C2—C1—C7	120.63 (14)	H10A—C10—H10B	108.1
C3—C2—C1	121.32 (15)	C10—C11—S1A	125.17 (13)
C3—C2—H2	119.3	C10—C11—C12	125.17 (13)
C1—C2—H2	119.3	C10—C11—C12A	123.64 (13)
C2—C3—C4	120.62 (15)	S1A—C11—C12A	110.77 (11)
C2—C3—H3	119.7	C10—C11—S1	123.64 (13)
C4—C3—H3	119.7	C12—C11—S1	110.77 (11)
C5—C4—C3	119.79 (15)	C13—C12—C11	103.59 (13)
C5—C4—H4	120.1	C13—C12—H12	128.2
C3—C4—H4	120.1	C11—C12—H12	128.2
C4—C5—C6	118.79 (15)	C13—S1A—C11	103.59 (13)
C4—C5—H5	120.6	C14—C13—C12	115.17 (18)
C6—C5—H5	120.6	C14—C13—S1A	115.17 (18)
C5—C6—C1	123.27 (15)	C14—C13—H13	122.4
C5—C6—N3	115.94 (14)	C12—C13—H13	122.4
C1—C6—N3	120.78 (14)	C13—C14—C12A	114.26 (16)
N1—C7—C1	118.69 (14)	C13—C14—S1	114.26 (16)
N1—C7—H7	120.7	C13—C14—H14	122.9
C1—C7—H7	120.7	S1—C14—H14	122.9
N2—C8—H8A	109.5	C7—N1—N2	118.03 (13)
N2—C8—H8B	109.5	N1—N2—C9	117.30 (12)
H8A—C8—H8B	109.5	N1—N2—C8	121.97 (13)
N2—C8—H8C	109.5	C9—N2—C8	120.73 (13)
H8A—C8—H8C	109.5	O2—N3—O3	123.62 (14)
H8B—C8—H8C	109.5	O2—N3—C6	118.85 (13)
O1—C9—N2	120.74 (14)	O3—N3—C6	117.50 (14)
O1—C9—C10	121.57 (14)	C14—S1—C11	95.85 (10)
N2—C9—C10	117.66 (14)	C14—C12A—C11	95.85 (10)
C11—C10—C9	110.52 (13)	C14—C12A—H12A	132.1
C11—C10—H10A	109.5	C11—C12A—H12A	132.1
C9—C10—H10A	109.5		
			
C6—C1—C2—C3	−1.6 (2)	C12A—C11—S1A—C13	−5.65 (15)
C7—C1—C2—C3	−178.31 (15)	C11—C12—C13—C14	6.2 (2)
C1—C2—C3—C4	1.6 (3)	C11—S1A—C13—C14	6.2 (2)
C2—C3—C4—C5	0.0 (3)	S1A—C13—C14—C12A	−4.3 (2)
C3—C4—C5—C6	−1.4 (2)	C12—C13—C14—S1	−4.3 (2)
C4—C5—C6—C1	1.3 (2)	C1—C7—N1—N2	179.10 (13)
C4—C5—C6—N3	−177.29 (14)	C7—N1—N2—C9	−175.84 (14)
C2—C1—C6—C5	0.2 (2)	C7—N1—N2—C8	3.2 (2)
C7—C1—C6—C5	176.78 (15)	O1—C9—N2—N1	176.75 (14)
C2—C1—C6—N3	178.71 (14)	C10—C9—N2—N1	−5.0 (2)
C7—C1—C6—N3	−4.7 (2)	O1—C9—N2—C8	−2.3 (2)
C6—C1—C7—N1	−176.15 (15)	C10—C9—N2—C8	175.95 (14)
C2—C1—C7—N1	0.3 (2)	C5—C6—N3—O2	135.41 (16)
O1—C9—C10—C11	96.54 (18)	C1—C6—N3—O2	−43.2 (2)
N2—C9—C10—C11	−81.73 (18)	C5—C6—N3—O3	−42.9 (2)
C9—C10—C11—S1A	−37.7 (2)	C1—C6—N3—O3	138.50 (16)
C9—C10—C11—C12	−37.7 (2)	C13—C14—S1—C11	0.41 (17)
C9—C10—C11—C12A	150.50 (13)	C10—C11—S1—C14	176.26 (14)
C9—C10—C11—S1	150.50 (13)	C12—C11—S1—C14	3.38 (13)
C10—C11—C12—C13	−178.39 (15)	C13—C14—C12A—C11	0.41 (17)
S1—C11—C12—C13	−5.65 (15)	C10—C11—C12A—C14	176.26 (14)
C10—C11—S1A—C13	−178.39 (15)	S1A—C11—C12A—C14	3.38 (13)

### (I) (E)-N-Methyl-N'-(2-nitrobenzylidene)-2-(thiophen-2-yl)acetohydrazide Hydrogen-bond geometry (Å, º)

Cg1 is the centroid of the S1/C11–C14 ring.

**Table:**

*D*—H···*A*	*D*—H	H···*A*	*D*···*A*	*D*—H···*A*
C8—H8*C*···O1^i^	0.98	2.49	3.293 (2)	139
C10—H10*A*···O3^ii^	0.99	2.55	3.386 (2)	142
C10—H10*B*···O1^iii^	0.99	2.52	3.439 (2)	154
C5—H5···*Cg*1^iv^	0.95	2.86	3.7212 (18)	151
C12—H12···*Cg*1^i^	0.95	2.85	3.5930 (13)	136

Symmetry codes: (i) *x*, −*y*+1/2, *z*−1/2; (ii) −*x*, −*y*+1, −*z*+1; (iii) *x*, −*y*+1/2, *z*+1/2; (iv) −*x*+1, −*y*+1, −*z*+1.

### (II) (E)-N-Methyl-N'-(3-nitrobenzylidene)-2-(thiophen-2-yl)acetohydrazide Crystal data

**Table:**

C_14_H_13_N_3_O_3_S	*F*(000) = 632
*M**_r_* = 303.33	*D*_x_ = 1.487 Mg m^−^^3^
Monoclinic, *P*2_1_/*n*	Mo *K*α radiation, λ = 0.71073 Å
*a* = 5.6629 (4) Å	Cell parameters from 9051 reflections
*b* = 15.6864 (11) Å	θ = 2.6–27.5°
*c* = 15.2842 (11) Å	µ = 0.25 mm^−^^1^
β = 93.3800 (18)°	*T* = 100 K
*V* = 1355.34 (17) Å^3^	Block, yellow
*Z* = 4	0.22 × 0.17 × 0.12 mm

### (II) (E)-N-Methyl-N'-(3-nitrobenzylidene)-2-(thiophen-2-yl)acetohydrazide Data collection

**Table:**

Rigaku Mercury CCD diffractometer	*R*_int_ = 0.031
ω scans	θ_max_ = 27.5°, θ_min_ = 2.6°
9365 measured reflections	*h* = −7→7
3110 independent reflections	*k* = −20→19
2757 reflections with *I* > 2σ(*I*)	*l* = −19→19

### (II) (E)-N-Methyl-N'-(3-nitrobenzylidene)-2-(thiophen-2-yl)acetohydrazide Refinement

**Table:**

Refinement on *F*^2^	0 restraints
Least-squares matrix: full	Hydrogen site location: inferred from neighbouring sites
*R*[*F*^2^ > 2σ(*F*^2^)] = 0.034	H-atom parameters constrained
*wR*(*F*^2^) = 0.096	*w* = 1/[σ^2^(*F*_o_^2^) + (0.0505*P*)^2^ + 0.4026*P*] where *P* = (*F*_o_^2^ + 2*F*_c_^2^)/3
*S* = 1.08	(Δ/σ)_max_ < 0.001
3110 reflections	Δρ_max_ = 0.30 e Å^−^^3^
191 parameters	Δρ_min_ = −0.28 e Å^−^^3^

### (II) (E)-N-Methyl-N'-(3-nitrobenzylidene)-2-(thiophen-2-yl)acetohydrazide Special details

**Table:**

Geometry. All esds (except the esd in the dihedral angle between two l.s. planes) are estimated using the full covariance matrix. The cell esds are taken into account individually in the estimation of esds in distances, angles and torsion angles; correlations between esds in cell parameters are only used when they are defined by crystal symmetry. An approximate (isotropic) treatment of cell esds is used for estimating esds involving l.s. planes.

### (II) (E)-N-Methyl-N'-(3-nitrobenzylidene)-2-(thiophen-2-yl)acetohydrazide Fractional atomic coordinates and isotropic or equivalent isotropic displacement parameters (Å^2^)

**Table:**

	*x*	*y*	*z*	*U*_iso_*/*U*_eq_	
C1	0.3945 (2)	0.25348 (8)	0.28563 (8)	0.0187 (2)	
C2	0.4786 (2)	0.33560 (8)	0.27161 (8)	0.0188 (2)	
H2	0.6256	0.3540	0.2983	0.023*	
C3	0.3425 (2)	0.38950 (8)	0.21792 (8)	0.0192 (3)	
C4	0.1240 (2)	0.36637 (9)	0.17844 (8)	0.0212 (3)	
H4	0.0333	0.4055	0.1430	0.025*	
C5	0.0432 (2)	0.28453 (9)	0.19246 (8)	0.0225 (3)	
H5	−0.1045	0.2667	0.1659	0.027*	
C6	0.1768 (2)	0.22833 (8)	0.24516 (8)	0.0210 (3)	
H6	0.1202	0.1721	0.2539	0.025*	
C7	0.5216 (2)	0.19343 (8)	0.34489 (8)	0.0198 (3)	
H7	0.4668	0.1365	0.3494	0.024*	
C8	0.7162 (2)	0.07490 (8)	0.45928 (9)	0.0225 (3)	
H8A	0.5539	0.0795	0.4777	0.034*	
H8B	0.8134	0.0441	0.5040	0.034*	
H8C	0.7160	0.0439	0.4036	0.034*	
C9	1.0134 (2)	0.18638 (9)	0.49611 (8)	0.0199 (3)	
C10	1.1004 (2)	0.27656 (8)	0.48044 (8)	0.0211 (3)	
H10A	1.2731	0.2785	0.4947	0.025*	
H10B	1.0732	0.2901	0.4174	0.025*	
C11	0.9841 (2)	0.34407 (8)	0.53279 (8)	0.0195 (3)	
C12	1.0854 (2)	0.38920 (9)	0.60202 (9)	0.0239 (3)	
H12	1.2409	0.3788	0.6265	0.029*	
C13	0.9330 (2)	0.45351 (9)	0.63371 (9)	0.0248 (3)	
H13	0.9760	0.4907	0.6810	0.030*	
C14	0.7188 (2)	0.45511 (9)	0.58811 (9)	0.0253 (3)	
H14	0.5950	0.4936	0.5998	0.030*	
N1	0.70671 (18)	0.21766 (7)	0.39066 (7)	0.0189 (2)	
N2	0.81324 (18)	0.15999 (7)	0.44806 (7)	0.0195 (2)	
N3	0.43337 (19)	0.47517 (7)	0.20197 (7)	0.0213 (2)	
O1	1.11711 (16)	0.13877 (6)	0.54872 (6)	0.0251 (2)	
O2	0.31572 (17)	0.52296 (6)	0.15251 (6)	0.0262 (2)	
O3	0.62386 (17)	0.49615 (7)	0.23846 (7)	0.0291 (2)	
S1	0.69978 (5)	0.37937 (2)	0.50723 (2)	0.02407 (11)	

### (II) (E)-N-Methyl-N'-(3-nitrobenzylidene)-2-(thiophen-2-yl)acetohydrazide Atomic displacement parameters (Å^2^)

**Table:**

	*U*^11^	*U*^22^	*U*^33^	*U*^12^	*U*^13^	*U*^23^
C1	0.0173 (5)	0.0210 (6)	0.0175 (5)	0.0019 (5)	−0.0020 (4)	−0.0014 (5)
C2	0.0153 (5)	0.0219 (6)	0.0186 (6)	0.0009 (5)	−0.0024 (4)	−0.0011 (5)
C3	0.0185 (6)	0.0195 (6)	0.0192 (6)	0.0004 (5)	−0.0011 (5)	−0.0015 (5)
C4	0.0183 (6)	0.0247 (6)	0.0199 (6)	0.0036 (5)	−0.0040 (5)	−0.0003 (5)
C5	0.0169 (6)	0.0278 (7)	0.0221 (6)	0.0001 (5)	−0.0051 (5)	−0.0025 (5)
C6	0.0191 (6)	0.0218 (6)	0.0217 (6)	−0.0014 (5)	−0.0029 (5)	−0.0016 (5)
C7	0.0184 (5)	0.0194 (6)	0.0212 (6)	−0.0004 (5)	−0.0023 (5)	0.0006 (5)
C8	0.0229 (6)	0.0184 (6)	0.0252 (6)	−0.0010 (5)	−0.0053 (5)	0.0022 (5)
C9	0.0157 (5)	0.0239 (6)	0.0199 (6)	0.0023 (5)	−0.0013 (4)	−0.0031 (5)
C10	0.0141 (5)	0.0250 (6)	0.0239 (6)	−0.0009 (5)	−0.0017 (4)	−0.0019 (5)
C11	0.0152 (5)	0.0207 (6)	0.0223 (6)	−0.0012 (5)	−0.0015 (4)	0.0018 (5)
C12	0.0182 (6)	0.0266 (7)	0.0265 (7)	−0.0015 (5)	−0.0017 (5)	−0.0023 (5)
C13	0.0217 (6)	0.0259 (7)	0.0264 (6)	−0.0010 (5)	−0.0028 (5)	−0.0039 (5)
C14	0.0232 (6)	0.0232 (7)	0.0292 (7)	0.0029 (5)	−0.0010 (5)	−0.0023 (5)
N1	0.0171 (5)	0.0199 (5)	0.0193 (5)	0.0022 (4)	−0.0025 (4)	0.0017 (4)
N2	0.0179 (5)	0.0184 (5)	0.0215 (5)	0.0005 (4)	−0.0055 (4)	0.0018 (4)
N3	0.0215 (5)	0.0212 (5)	0.0208 (5)	0.0011 (4)	−0.0029 (4)	−0.0009 (4)
O1	0.0215 (4)	0.0274 (5)	0.0254 (5)	0.0037 (4)	−0.0077 (4)	0.0008 (4)
O2	0.0284 (5)	0.0215 (5)	0.0280 (5)	0.0056 (4)	−0.0061 (4)	0.0028 (4)
O3	0.0263 (5)	0.0283 (5)	0.0312 (5)	−0.0069 (4)	−0.0104 (4)	0.0028 (4)
S1	0.01743 (17)	0.02608 (19)	0.02791 (19)	0.00234 (12)	−0.00532 (13)	−0.00320 (13)

### (II) (E)-N-Methyl-N'-(3-nitrobenzylidene)-2-(thiophen-2-yl)acetohydrazide Geometric parameters (Å, º)

**Table:**

C1—C2	1.3943 (18)	C9—O1	1.2220 (16)
C1—C6	1.4027 (16)	C9—N2	1.3776 (15)
C1—C7	1.4659 (17)	C9—C10	1.5215 (18)
C2—C3	1.3811 (17)	C10—C11	1.5025 (18)
C2—H2	0.9500	C10—H10A	0.9900
C3—C4	1.3923 (17)	C10—H10B	0.9900
C3—N3	1.4645 (17)	C11—C12	1.3705 (18)
C4—C5	1.3839 (19)	C11—S1	1.7256 (12)
C4—H4	0.9500	C12—C13	1.4306 (19)
C5—C6	1.3883 (18)	C12—H12	0.9500
C5—H5	0.9500	C13—C14	1.3630 (18)
C6—H6	0.9500	C13—H13	0.9500
C7—N1	1.2829 (16)	C14—S1	1.7134 (14)
C7—H7	0.9500	C14—H14	0.9500
C8—N2	1.4573 (16)	N1—N2	1.3747 (14)
C8—H8A	0.9800	N3—O3	1.2297 (14)
C8—H8B	0.9800	N3—O2	1.2321 (14)
C8—H8C	0.9800		
			
C2—C1—C6	119.43 (11)	O1—C9—C10	121.63 (11)
C2—C1—C7	121.93 (11)	N2—C9—C10	117.35 (11)
C6—C1—C7	118.58 (12)	C11—C10—C9	114.54 (11)
C3—C2—C1	118.24 (11)	C11—C10—H10A	108.6
C3—C2—H2	120.9	C9—C10—H10A	108.6
C1—C2—H2	120.9	C11—C10—H10B	108.6
C2—C3—C4	123.26 (12)	C9—C10—H10B	108.6
C2—C3—N3	118.13 (11)	H10A—C10—H10B	107.6
C4—C3—N3	118.62 (11)	C12—C11—C10	126.76 (11)
C5—C4—C3	117.93 (12)	C12—C11—S1	110.59 (10)
C5—C4—H4	121.0	C10—C11—S1	122.55 (9)
C3—C4—H4	121.0	C11—C12—C13	113.09 (12)
C4—C5—C6	120.32 (11)	C11—C12—H12	123.5
C4—C5—H5	119.8	C13—C12—H12	123.5
C6—C5—H5	119.8	C14—C13—C12	112.11 (12)
C5—C6—C1	120.80 (12)	C14—C13—H13	123.9
C5—C6—H6	119.6	C12—C13—H13	123.9
C1—C6—H6	119.6	C13—C14—S1	111.86 (11)
N1—C7—C1	120.14 (12)	C13—C14—H14	124.1
N1—C7—H7	119.9	S1—C14—H14	124.1
C1—C7—H7	119.9	C7—N1—N2	117.82 (11)
N2—C8—H8A	109.5	N1—N2—C9	117.31 (11)
N2—C8—H8B	109.5	N1—N2—C8	121.56 (10)
H8A—C8—H8B	109.5	C9—N2—C8	121.11 (10)
N2—C8—H8C	109.5	O3—N3—O2	122.93 (11)
H8A—C8—H8C	109.5	O3—N3—C3	118.52 (10)
H8B—C8—H8C	109.5	O2—N3—C3	118.55 (10)
O1—C9—N2	121.03 (12)	C14—S1—C11	92.35 (6)
			
C6—C1—C2—C3	0.08 (18)	S1—C11—C12—C13	−0.83 (15)
C7—C1—C2—C3	−177.02 (12)	C11—C12—C13—C14	0.49 (18)
C1—C2—C3—C4	1.15 (19)	C12—C13—C14—S1	0.08 (16)
C1—C2—C3—N3	−178.86 (11)	C1—C7—N1—N2	177.17 (11)
C2—C3—C4—C5	−1.5 (2)	C7—N1—N2—C9	179.26 (11)
N3—C3—C4—C5	178.49 (12)	C7—N1—N2—C8	−2.03 (17)
C3—C4—C5—C6	0.64 (19)	O1—C9—N2—N1	179.67 (11)
C4—C5—C6—C1	0.5 (2)	C10—C9—N2—N1	−0.78 (16)
C2—C1—C6—C5	−0.90 (19)	O1—C9—N2—C8	0.95 (19)
C7—C1—C6—C5	176.30 (12)	C10—C9—N2—C8	−179.50 (11)
C2—C1—C7—N1	5.17 (19)	C2—C3—N3—O3	−1.60 (18)
C6—C1—C7—N1	−171.96 (12)	C4—C3—N3—O3	178.39 (12)
O1—C9—C10—C11	−95.28 (14)	C2—C3—N3—O2	178.30 (11)
N2—C9—C10—C11	85.18 (14)	C4—C3—N3—O2	−1.70 (17)
C9—C10—C11—C12	108.80 (15)	C13—C14—S1—C11	−0.47 (12)
C9—C10—C11—S1	−75.20 (14)	C12—C11—S1—C14	0.74 (11)
C10—C11—C12—C13	175.57 (13)	C10—C11—S1—C14	−175.84 (11)

### (II) (E)-N-Methyl-N'-(3-nitrobenzylidene)-2-(thiophen-2-yl)acetohydrazide Hydrogen-bond geometry (Å, º)

**Table:**

*D*—H···*A*	*D*—H	H···*A*	*D*···*A*	*D*—H···*A*
C7—H7···O2^i^	0.95	2.39	3.2879 (16)	157
C8—H8*B*···O2^ii^	0.98	2.50	3.3468 (16)	144
C8—H8*C*···O3^iii^	0.98	2.52	3.4356 (17)	156
C13—H13···O3^iv^	0.95	2.52	3.1874 (16)	127

Symmetry codes: (i) −*x*+1/2, *y*−1/2, −*z*+1/2; (ii) *x*+1/2, −*y*+1/2, *z*+1/2; (iii) −*x*+3/2, *y*−1/2, −*z*+1/2; (iv) −*x*+2, −*y*+1, −*z*+1.

### (III) (E)-N-Methyl-N'-(4-nitrobenzylidene)-2-(thiophen-2-yl)acetohydrazide Crystal data

**Table:**

C_14_H_13_N_3_O_3_S	*Z* = 4
*M**_r_* = 303.33	*F*(000) = 632
Triclinic, *P*1	*D*_x_ = 1.461 Mg m^−^^3^
*a* = 6.1893 (4) Å	Mo *K*α radiation, λ = 0.71073 Å
*b* = 12.9177 (9) Å	Cell parameters from 15464 reflections
*c* = 17.3828 (12) Å	θ = 3.2–27.6°
α = 93.995 (7)°	µ = 0.25 mm^−^^1^
β = 90.386 (6)°	*T* = 100 K
γ = 95.963 (7)°	Cut block, yellow
*V* = 1378.77 (16) Å^3^	0.20 × 0.18 × 0.16 mm

### (III) (E)-N-Methyl-N'-(4-nitrobenzylidene)-2-(thiophen-2-yl)acetohydrazide Data collection

**Table:**

Rigaku Mercury CCD diffractometer	*R*_int_ = 0.078
ω scans	θ_max_ = 27.5°, θ_min_ = 3.2°
18534 measured reflections	*h* = −7→8
6279 independent reflections	*k* = −16→16
4868 reflections with *I* > 2σ(*I*)	*l* = −22→22

### (III) (E)-N-Methyl-N'-(4-nitrobenzylidene)-2-(thiophen-2-yl)acetohydrazide Refinement

**Table:**

Refinement on *F*^2^	0 restraints
Least-squares matrix: full	Hydrogen site location: inferred from neighbouring sites
*R*[*F*^2^ > 2σ(*F*^2^)] = 0.058	H-atom parameters constrained
*wR*(*F*^2^) = 0.166	*w* = 1/[σ^2^(*F*_o_^2^) + (0.080*P*)^2^ + 0.2939*P*] where *P* = (*F*_o_^2^ + 2*F*_c_^2^)/3
*S* = 1.10	(Δ/σ)_max_ < 0.001
6279 reflections	Δρ_max_ = 0.67 e Å^−^^3^
383 parameters	Δρ_min_ = −0.61 e Å^−^^3^

### (III) (E)-N-Methyl-N'-(4-nitrobenzylidene)-2-(thiophen-2-yl)acetohydrazide Special details

**Table:**

Geometry. All esds (except the esd in the dihedral angle between two l.s. planes) are estimated using the full covariance matrix. The cell esds are taken into account individually in the estimation of esds in distances, angles and torsion angles; correlations between esds in cell parameters are only used when they are defined by crystal symmetry. An approximate (isotropic) treatment of cell esds is used for estimating esds involving l.s. planes.

### (III) (E)-N-Methyl-N'-(4-nitrobenzylidene)-2-(thiophen-2-yl)acetohydrazide Fractional atomic coordinates and isotropic or equivalent isotropic displacement parameters (Å^2^)

**Table:**

	*x*	*y*	*z*	*U*_iso_*/*U*_eq_	Occ. (<1)
C1	0.3947 (3)	0.10043 (14)	0.12117 (11)	0.0207 (4)	
C2	0.3601 (3)	0.01607 (15)	0.16786 (12)	0.0252 (4)	
H2	0.2397	0.0115	0.2015	0.030*	
C3	0.5005 (4)	−0.06029 (15)	0.16500 (12)	0.0271 (5)	
H3	0.4784	−0.1176	0.1965	0.033*	
C4	0.6747 (3)	−0.05157 (14)	0.11517 (12)	0.0226 (4)	
C5	0.7135 (3)	0.03028 (14)	0.06866 (11)	0.0218 (4)	
H5	0.8345	0.0344	0.0353	0.026*	
C6	0.5712 (3)	0.10625 (14)	0.07199 (11)	0.0210 (4)	
H6	0.5945	0.1632	0.0402	0.025*	
C7	0.2465 (3)	0.18208 (14)	0.12183 (11)	0.0213 (4)	
H7	0.2660	0.2356	0.0869	0.026*	
C8	−0.0467 (4)	0.32516 (15)	0.10527 (12)	0.0255 (4)	
H8A	−0.0462	0.2846	0.0555	0.038*	
H8B	0.0845	0.3748	0.1104	0.038*	
H8C	−0.1755	0.3634	0.1081	0.038*	
C9	−0.2093 (3)	0.25364 (14)	0.22263 (11)	0.0220 (4)	
C10	−0.2139 (3)	0.17041 (14)	0.28040 (12)	0.0235 (4)	
H10A	−0.3660	0.1502	0.2950	0.028*	
H10B	−0.1553	0.1077	0.2562	0.028*	
C11	−0.0820 (3)	0.20882 (14)	0.35164 (11)	0.0223 (4)	
C12	−0.1921 (3)	0.21642 (9)	0.43327 (7)	0.0459 (5)	0.673 (3)
H12	−0.3381	0.1994	0.4483	0.055*	0.673 (3)
S1A	−0.1921 (3)	0.21642 (9)	0.43327 (7)	0.0459 (5)	0.327 (3)
C13	0.0169 (4)	0.26112 (17)	0.48174 (13)	0.0325 (5)	
H13	0.0111	0.2762	0.5359	0.039*	
C14	0.2039 (4)	0.27710 (16)	0.44338 (13)	0.0318 (5)	
H14	0.3361	0.3048	0.4687	0.038*	
N1	0.0911 (3)	0.18039 (12)	0.16982 (9)	0.0207 (4)	
N2	−0.0509 (3)	0.25508 (12)	0.16718 (10)	0.0214 (4)	
N3	0.8213 (3)	−0.13383 (13)	0.11193 (11)	0.0282 (4)	
O1	−0.3402 (2)	0.31816 (11)	0.22537 (9)	0.0288 (3)	
O2	0.9584 (2)	−0.13473 (11)	0.06134 (9)	0.0323 (4)	
O3	0.7993 (3)	−0.19851 (13)	0.16061 (11)	0.0489 (5)	
S1	0.18984 (12)	0.24543 (5)	0.34894 (4)	0.0275 (2)	0.673 (3)
C12A	0.18984 (12)	0.24543 (5)	0.34894 (4)	0.0275 (2)	0.327 (3)
H12A	0.2936	0.2471	0.3089	0.033*	0.327 (3)
C15	−0.1307 (3)	0.45425 (14)	0.76584 (11)	0.0204 (4)	
C16	−0.3149 (3)	0.50824 (13)	0.77270 (11)	0.0211 (4)	
H16	−0.3395	0.5585	0.7371	0.025*	
C17	−0.4611 (3)	0.48896 (14)	0.83085 (11)	0.0213 (4)	
H17	−0.5869	0.5252	0.8355	0.026*	
C18	−0.4209 (3)	0.41575 (14)	0.88233 (11)	0.0215 (4)	
C19	−0.2380 (3)	0.36200 (15)	0.87768 (12)	0.0237 (4)	
H19	−0.2135	0.3122	0.9136	0.028*	
C20	−0.0932 (3)	0.38266 (14)	0.81973 (12)	0.0228 (4)	
H20	0.0344	0.3477	0.8163	0.027*	
C21	0.0221 (3)	0.47259 (14)	0.70286 (11)	0.0211 (4)	
H21	0.0129	0.5292	0.6713	0.025*	
C22	0.3281 (4)	0.52050 (16)	0.59201 (12)	0.0302 (5)	
H22A	0.3299	0.5823	0.6281	0.045*	
H22B	0.2012	0.5167	0.5575	0.045*	
H22C	0.4609	0.5252	0.5615	0.045*	
C23	0.4675 (3)	0.35588 (14)	0.62403 (11)	0.0216 (4)	
C24	0.4422 (3)	0.26350 (14)	0.67397 (12)	0.0235 (4)	
H24A	0.4741	0.2890	0.7283	0.028*	
H24B	0.2893	0.2322	0.6708	0.028*	
C25	0.5867 (4)	0.18068 (14)	0.65165 (12)	0.0247 (4)	
C26	0.8082 (3)	0.17391 (11)	0.67653 (9)	0.0332 (5)	0.832 (3)
H26	0.8933	0.2246	0.7093	0.040*	0.832 (3)
S2A	0.8082 (3)	0.17391 (11)	0.67653 (9)	0.0332 (5)	0.168 (3)
C27	0.8783 (4)	0.07846 (18)	0.64370 (14)	0.0363 (5)	
H27	1.0182	0.0579	0.6536	0.044*	
C28	0.7311 (5)	0.02082 (17)	0.59807 (15)	0.0452 (7)	
H28	0.7552	−0.0441	0.5722	0.054*	
N4	0.1678 (3)	0.41024 (12)	0.69164 (9)	0.0204 (4)	
N5	0.3168 (3)	0.42739 (12)	0.63490 (9)	0.0218 (4)	
N6	−0.5754 (3)	0.39473 (13)	0.94419 (10)	0.0249 (4)	
O4	0.6102 (2)	0.36793 (11)	0.57669 (8)	0.0279 (3)	
O5	−0.7216 (2)	0.45094 (11)	0.95462 (9)	0.0296 (3)	
O6	−0.5498 (3)	0.32116 (12)	0.98361 (10)	0.0373 (4)	
S2	0.49917 (12)	0.07608 (5)	0.59164 (4)	0.0380 (3)	0.832 (3)
C26A	0.49917 (12)	0.07608 (5)	0.59164 (4)	0.0380 (3)	0.168 (3)
H26A	0.3665	0.0559	0.5642	0.046*	0.168 (3)

### (III) (E)-N-Methyl-N'-(4-nitrobenzylidene)-2-(thiophen-2-yl)acetohydrazide Atomic displacement parameters (Å^2^)

**Table:**

	*U*^11^	*U*^22^	*U*^33^	*U*^12^	*U*^13^	*U*^23^
C1	0.0237 (10)	0.0193 (8)	0.0186 (9)	0.0028 (7)	−0.0027 (8)	−0.0026 (7)
C2	0.0270 (11)	0.0258 (9)	0.0235 (11)	0.0057 (8)	0.0055 (9)	0.0022 (8)
C3	0.0359 (12)	0.0220 (9)	0.0246 (11)	0.0063 (8)	0.0024 (9)	0.0041 (8)
C4	0.0258 (10)	0.0205 (9)	0.0218 (10)	0.0078 (8)	−0.0022 (8)	−0.0041 (7)
C5	0.0230 (10)	0.0210 (9)	0.0204 (10)	0.0014 (7)	−0.0008 (8)	−0.0040 (7)
C6	0.0253 (10)	0.0167 (8)	0.0202 (10)	0.0009 (7)	−0.0023 (8)	−0.0014 (7)
C7	0.0246 (10)	0.0188 (8)	0.0203 (10)	0.0017 (7)	−0.0008 (8)	0.0004 (7)
C8	0.0341 (12)	0.0223 (9)	0.0207 (10)	0.0079 (8)	−0.0023 (9)	−0.0008 (8)
C9	0.0240 (10)	0.0216 (9)	0.0197 (10)	0.0035 (8)	−0.0025 (8)	−0.0049 (7)
C10	0.0255 (10)	0.0202 (9)	0.0245 (11)	0.0035 (8)	0.0015 (9)	−0.0025 (8)
C11	0.0297 (11)	0.0175 (8)	0.0203 (10)	0.0056 (8)	0.0037 (8)	0.0012 (7)
C12	0.0713 (10)	0.0336 (7)	0.0324 (7)	0.0083 (6)	−0.0152 (7)	−0.0034 (5)
S1A	0.0713 (10)	0.0336 (7)	0.0324 (7)	0.0083 (6)	−0.0152 (7)	−0.0034 (5)
C13	0.0431 (13)	0.0343 (11)	0.0211 (11)	0.0075 (10)	0.0028 (10)	0.0031 (9)
C14	0.0384 (13)	0.0269 (10)	0.0296 (12)	0.0031 (9)	0.0000 (10)	−0.0002 (9)
N1	0.0234 (9)	0.0196 (7)	0.0191 (8)	0.0052 (6)	−0.0032 (7)	−0.0040 (6)
N2	0.0245 (9)	0.0209 (7)	0.0193 (8)	0.0065 (6)	−0.0011 (7)	−0.0016 (6)
N3	0.0332 (10)	0.0259 (8)	0.0266 (10)	0.0110 (7)	0.0013 (8)	−0.0009 (7)
O1	0.0329 (8)	0.0276 (7)	0.0272 (8)	0.0124 (6)	0.0013 (7)	−0.0037 (6)
O2	0.0324 (9)	0.0300 (7)	0.0359 (9)	0.0111 (6)	0.0100 (7)	−0.0013 (7)
O3	0.0667 (13)	0.0449 (10)	0.0436 (11)	0.0335 (9)	0.0174 (9)	0.0205 (8)
S1	0.0339 (4)	0.0253 (4)	0.0227 (4)	0.0008 (3)	−0.0028 (3)	0.0004 (3)
C12A	0.0339 (4)	0.0253 (4)	0.0227 (4)	0.0008 (3)	−0.0028 (3)	0.0004 (3)
C15	0.0249 (10)	0.0169 (8)	0.0184 (10)	0.0020 (7)	−0.0025 (8)	−0.0046 (7)
C16	0.0288 (10)	0.0152 (8)	0.0194 (10)	0.0040 (7)	−0.0029 (8)	−0.0002 (7)
C17	0.0243 (10)	0.0190 (8)	0.0206 (10)	0.0051 (7)	−0.0019 (8)	−0.0038 (7)
C18	0.0262 (10)	0.0197 (8)	0.0178 (10)	0.0021 (8)	0.0016 (8)	−0.0043 (7)
C19	0.0304 (11)	0.0232 (9)	0.0186 (10)	0.0076 (8)	0.0003 (8)	0.0011 (7)
C20	0.0252 (10)	0.0227 (9)	0.0214 (10)	0.0091 (8)	0.0005 (8)	−0.0026 (7)
C21	0.0275 (11)	0.0184 (8)	0.0171 (9)	0.0032 (7)	−0.0017 (8)	−0.0003 (7)
C22	0.0442 (13)	0.0246 (10)	0.0232 (11)	0.0080 (9)	0.0080 (10)	0.0046 (8)
C23	0.0264 (11)	0.0205 (9)	0.0167 (9)	0.0013 (8)	−0.0009 (8)	−0.0040 (7)
C24	0.0294 (11)	0.0211 (9)	0.0203 (10)	0.0044 (8)	0.0043 (8)	0.0000 (7)
C25	0.0356 (12)	0.0198 (9)	0.0190 (10)	0.0045 (8)	0.0075 (9)	0.0000 (7)
C26	0.0387 (10)	0.0257 (7)	0.0347 (9)	0.0027 (6)	0.0045 (7)	−0.0006 (6)
S2A	0.0387 (10)	0.0257 (7)	0.0347 (9)	0.0027 (6)	0.0045 (7)	−0.0006 (6)
C27	0.0370 (13)	0.0357 (12)	0.0393 (14)	0.0140 (10)	0.0097 (11)	0.0088 (10)
C28	0.079 (2)	0.0235 (10)	0.0347 (14)	0.0187 (12)	0.0072 (13)	−0.0043 (9)
N4	0.0248 (9)	0.0193 (7)	0.0160 (8)	0.0004 (6)	0.0015 (7)	−0.0036 (6)
N5	0.0284 (9)	0.0202 (7)	0.0167 (8)	0.0032 (7)	0.0041 (7)	−0.0002 (6)
N6	0.0270 (9)	0.0254 (8)	0.0218 (9)	0.0028 (7)	0.0010 (7)	−0.0011 (7)
O4	0.0335 (8)	0.0285 (7)	0.0224 (8)	0.0056 (6)	0.0096 (7)	0.0022 (6)
O5	0.0285 (8)	0.0346 (8)	0.0266 (8)	0.0100 (6)	0.0057 (6)	−0.0021 (6)
O6	0.0451 (10)	0.0363 (8)	0.0334 (9)	0.0107 (7)	0.0114 (8)	0.0138 (7)
S2	0.0456 (5)	0.0264 (3)	0.0403 (4)	0.0086 (3)	−0.0115 (3)	−0.0154 (3)
C26A	0.0456 (5)	0.0264 (3)	0.0403 (4)	0.0086 (3)	−0.0115 (3)	−0.0154 (3)

### (III) (E)-N-Methyl-N'-(4-nitrobenzylidene)-2-(thiophen-2-yl)acetohydrazide Geometric parameters (Å, º)

**Table:**

C1—C6	1.391 (3)	C15—C20	1.396 (3)
C1—C2	1.403 (3)	C15—C16	1.398 (3)
C1—C7	1.467 (3)	C15—C21	1.467 (3)
C2—C3	1.379 (3)	C16—C17	1.380 (3)
C2—H2	0.9500	C16—H16	0.9500
C3—C4	1.388 (3)	C17—C18	1.386 (3)
C3—H3	0.9500	C17—H17	0.9500
C4—C5	1.378 (3)	C18—C19	1.388 (3)
C4—N3	1.466 (2)	C18—N6	1.464 (3)
C5—C6	1.384 (3)	C19—C20	1.375 (3)
C5—H5	0.9500	C19—H19	0.9500
C6—H6	0.9500	C20—H20	0.9500
C7—N1	1.277 (3)	C21—N4	1.277 (2)
C7—H7	0.9500	C21—H21	0.9500
C8—N2	1.452 (3)	C22—N5	1.454 (2)
C8—H8A	0.9800	C22—H22A	0.9800
C8—H8B	0.9800	C22—H22B	0.9800
C8—H8C	0.9800	C22—H22C	0.9800
C9—O1	1.220 (2)	C23—O4	1.217 (2)
C9—N2	1.380 (3)	C23—N5	1.384 (2)
C9—C10	1.520 (3)	C23—C24	1.520 (3)
C10—C11	1.504 (3)	C24—C25	1.497 (3)
C10—H10A	0.9900	C24—H24A	0.9900
C10—H10B	0.9900	C24—H24B	0.9900
C11—S1A	1.579 (2)	C25—S2A	1.448 (3)
C11—C12	1.579 (2)	C25—C26	1.448 (3)
C11—C12A	1.702 (2)	C25—C26A	1.689 (2)
C11—S1	1.702 (2)	C25—S2	1.689 (2)
C12—C13	1.575 (3)	C26—C27	1.432 (3)
C12—H12	0.9500	C26—H26	0.9500
S1A—C13	1.575 (3)	S2A—C27	1.432 (3)
C13—C14	1.344 (3)	C27—C28	1.334 (4)
C13—H13	0.9500	C27—H27	0.9500
C14—C12A	1.663 (2)	C28—C26A	1.675 (3)
C14—S1	1.663 (2)	C28—S2	1.675 (3)
C14—H14	0.9500	C28—H28	0.9500
N1—N2	1.373 (2)	N4—N5	1.368 (2)
N3—O2	1.227 (2)	N6—O5	1.224 (2)
N3—O3	1.228 (2)	N6—O6	1.232 (2)
C12A—H12A	0.9500	C26A—H26A	0.9500
			
C6—C1—C2	119.49 (18)	C20—C15—C16	119.30 (18)
C6—C1—C7	118.95 (17)	C20—C15—C21	120.19 (18)
C2—C1—C7	121.55 (18)	C16—C15—C21	120.51 (17)
C3—C2—C1	120.15 (19)	C17—C16—C15	120.39 (17)
C3—C2—H2	119.9	C17—C16—H16	119.8
C1—C2—H2	119.9	C15—C16—H16	119.8
C2—C3—C4	118.57 (18)	C16—C17—C18	118.71 (18)
C2—C3—H3	120.7	C16—C17—H17	120.6
C4—C3—H3	120.7	C18—C17—H17	120.6
C5—C4—C3	122.75 (18)	C17—C18—C19	122.23 (18)
C5—C4—N3	119.17 (18)	C17—C18—N6	119.07 (17)
C3—C4—N3	118.08 (17)	C19—C18—N6	118.70 (17)
C4—C5—C6	118.10 (18)	C20—C19—C18	118.32 (18)
C4—C5—H5	121.0	C20—C19—H19	120.8
C6—C5—H5	121.0	C18—C19—H19	120.8
C5—C6—C1	120.95 (17)	C19—C20—C15	121.02 (18)
C5—C6—H6	119.5	C19—C20—H20	119.5
C1—C6—H6	119.5	C15—C20—H20	119.5
N1—C7—C1	119.28 (17)	N4—C21—C15	118.09 (17)
N1—C7—H7	120.4	N4—C21—H21	121.0
C1—C7—H7	120.4	C15—C21—H21	121.0
N2—C8—H8A	109.5	N5—C22—H22A	109.5
N2—C8—H8B	109.5	N5—C22—H22B	109.5
H8A—C8—H8B	109.5	H22A—C22—H22B	109.5
N2—C8—H8C	109.5	N5—C22—H22C	109.5
H8A—C8—H8C	109.5	H22A—C22—H22C	109.5
H8B—C8—H8C	109.5	H22B—C22—H22C	109.5
O1—C9—N2	120.65 (18)	O4—C23—N5	120.73 (17)
O1—C9—C10	121.28 (18)	O4—C23—C24	123.10 (17)
N2—C9—C10	118.07 (16)	N5—C23—C24	116.17 (17)
C11—C10—C9	111.36 (15)	C25—C24—C23	113.87 (17)
C11—C10—H10A	109.4	C25—C24—H24A	108.8
C9—C10—H10A	109.4	C23—C24—H24A	108.8
C11—C10—H10B	109.4	C25—C24—H24B	108.8
C9—C10—H10B	109.4	C23—C24—H24B	108.8
H10A—C10—H10B	108.0	H24A—C24—H24B	107.7
C10—C11—S1A	120.81 (16)	S2A—C25—C24	128.30 (17)
C10—C11—C12	120.81 (16)	C26—C25—C24	128.30 (17)
C10—C11—C12A	122.36 (15)	S2A—C25—C26A	110.16 (13)
S1A—C11—C12A	116.83 (13)	C24—C25—C26A	121.52 (16)
C10—C11—S1	122.36 (15)	C26—C25—S2	110.16 (13)
C12—C11—S1	116.83 (13)	C24—C25—S2	121.52 (16)
C13—C12—C11	97.55 (14)	C27—C26—C25	108.93 (16)
C13—C12—H12	131.2	C27—C26—H26	125.5
C11—C12—H12	131.2	C25—C26—H26	125.5
C13—S1A—C11	97.55 (14)	C27—S2A—C25	108.93 (16)
C14—C13—C12	117.32 (19)	C28—C27—C26	114.6 (2)
C14—C13—S1A	117.32 (19)	C28—C27—S2A	114.6 (2)
C14—C13—H13	121.3	C28—C27—H27	122.7
C12—C13—H13	121.3	C26—C27—H27	122.7
C13—C14—C12A	115.93 (18)	C27—C28—C26A	112.26 (17)
C13—C14—S1	115.93 (18)	C27—C28—S2	112.26 (17)
C13—C14—H14	122.0	C27—C28—H28	123.9
S1—C14—H14	122.0	S2—C28—H28	123.9
C7—N1—N2	118.20 (16)	C21—N4—N5	119.36 (16)
N1—N2—C9	116.20 (16)	N4—N5—C23	116.86 (15)
N1—N2—C8	121.72 (16)	N4—N5—C22	121.80 (16)
C9—N2—C8	121.84 (16)	C23—N5—C22	121.17 (17)
O2—N3—O3	123.55 (17)	O5—N6—O6	123.34 (18)
O2—N3—C4	118.83 (17)	O5—N6—C18	118.89 (16)
O3—N3—C4	117.62 (18)	O6—N6—C18	117.76 (17)
C14—S1—C11	92.35 (11)	C28—S2—C25	94.06 (11)
C14—C12A—C11	92.35 (11)	C28—C26A—C25	94.06 (11)
C14—C12A—H12A	133.8	C28—C26A—H26A	133.0
C11—C12A—H12A	133.8	C25—C26A—H26A	133.0
			
C6—C1—C2—C3	0.1 (3)	C20—C15—C16—C17	−1.7 (3)
C7—C1—C2—C3	178.96 (18)	C21—C15—C16—C17	178.39 (16)
C1—C2—C3—C4	−0.1 (3)	C15—C16—C17—C18	0.4 (3)
C2—C3—C4—C5	0.2 (3)	C16—C17—C18—C19	0.6 (3)
C2—C3—C4—N3	−179.27 (18)	C16—C17—C18—N6	−179.94 (16)
C3—C4—C5—C6	−0.3 (3)	C17—C18—C19—C20	−0.1 (3)
N3—C4—C5—C6	179.15 (17)	N6—C18—C19—C20	−179.61 (17)
C4—C5—C6—C1	0.3 (3)	C18—C19—C20—C15	−1.3 (3)
C2—C1—C6—C5	−0.2 (3)	C16—C15—C20—C19	2.2 (3)
C7—C1—C6—C5	−179.11 (17)	C21—C15—C20—C19	−177.92 (17)
C6—C1—C7—N1	−176.17 (17)	C20—C15—C21—N4	11.1 (3)
C2—C1—C7—N1	5.0 (3)	C16—C15—C21—N4	−169.05 (17)
O1—C9—C10—C11	−87.5 (2)	O4—C23—C24—C25	−8.7 (3)
N2—C9—C10—C11	91.7 (2)	N5—C23—C24—C25	171.09 (17)
C9—C10—C11—S1A	118.60 (17)	C23—C24—C25—S2A	87.0 (2)
C9—C10—C11—C12	118.60 (17)	C23—C24—C25—C26	87.0 (2)
C9—C10—C11—C12A	−61.7 (2)	C23—C24—C25—C26A	−94.5 (2)
C9—C10—C11—S1	−61.7 (2)	C23—C24—C25—S2	−94.5 (2)
C10—C11—C12—C13	−179.11 (16)	C24—C25—C26—C27	176.91 (19)
S1—C11—C12—C13	1.14 (15)	S2—C25—C26—C27	−1.77 (19)
C10—C11—S1A—C13	−179.11 (16)	C24—C25—S2A—C27	176.91 (19)
C12A—C11—S1A—C13	1.14 (15)	C26A—C25—S2A—C27	−1.77 (19)
C11—C12—C13—C14	−0.1 (2)	C25—C26—C27—C28	1.4 (3)
C11—S1A—C13—C14	−0.1 (2)	C25—S2A—C27—C28	1.4 (3)
S1A—C13—C14—C12A	−0.9 (3)	S2A—C27—C28—C26A	−0.3 (3)
C12—C13—C14—S1	−0.9 (3)	C26—C27—C28—S2	−0.3 (3)
C1—C7—N1—N2	−177.33 (15)	C15—C21—N4—N5	−177.94 (15)
C7—N1—N2—C9	−176.72 (16)	C21—N4—N5—C23	−178.41 (16)
C7—N1—N2—C8	8.8 (3)	C21—N4—N5—C22	6.3 (3)
O1—C9—N2—N1	178.83 (16)	O4—C23—N5—N4	−177.20 (16)
C10—C9—N2—N1	−0.5 (2)	C24—C23—N5—N4	3.0 (2)
O1—C9—N2—C8	−6.7 (3)	O4—C23—N5—C22	−1.9 (3)
C10—C9—N2—C8	174.00 (16)	C24—C23—N5—C22	178.24 (17)
C5—C4—N3—O2	−8.9 (3)	C17—C18—N6—O5	−8.7 (3)
C3—C4—N3—O2	170.54 (18)	C19—C18—N6—O5	170.78 (17)
C5—C4—N3—O3	171.05 (19)	C17—C18—N6—O6	171.80 (17)
C3—C4—N3—O3	−9.5 (3)	C19—C18—N6—O6	−8.7 (3)
C13—C14—S1—C11	1.36 (18)	C27—C28—S2—C25	−0.6 (2)
C10—C11—S1—C14	178.76 (16)	C26—C25—S2—C28	1.42 (16)
C12—C11—S1—C14	−1.49 (14)	C24—C25—S2—C28	−177.37 (18)
C13—C14—C12A—C11	1.36 (18)	C27—C28—C26A—C25	−0.6 (2)
C10—C11—C12A—C14	178.76 (16)	S2A—C25—C26A—C28	1.42 (16)
S1A—C11—C12A—C14	−1.49 (14)	C24—C25—C26A—C28	−177.37 (18)

### (III) (E)-N-Methyl-N'-(4-nitrobenzylidene)-2-(thiophen-2-yl)acetohydrazide Hydrogen-bond geometry (Å, º)

Cg6 is the centroid of the C15–C20 ring.

**Table:**

*D*—H···*A*	*D*—H	H···*A*	*D*···*A*	*D*—H···*A*
C5—H5···O2^i^	0.95	2.48	3.312 (3)	147
C6—H6···O6^ii^	0.95	2.56	3.412 (2)	149
C7—H7···O6^ii^	0.95	2.41	3.281 (3)	153
C14—H14···O4	0.95	2.55	3.464 (3)	160
C17—H17···O1^iii^	0.95	2.43	3.104 (2)	128
C20—H20···O3^iv^	0.95	2.33	3.176 (2)	147
C8—H8*B*···*Cg*6^v^	0.98	2.77	3.634 (2)	147
C24—H24*A*···*Cg*6^vi^	0.98	2.77	3.628 (2)	145

Symmetry codes: (i) −*x*+2, −*y*, −*z*; (ii) *x*+1, *y*, *z*−1; (iii) −*x*−1, −*y*+1, −*z*+1; (iv) −*x*+1, −*y*, −*z*+1; (v) −*x*, −*y*+1, −*z*+1; (vi) *x*+1, *y*, *z*.
